# Epidemiology, causes, evolution and outcome in a single-center cohort of 1116 critically ill patients with hypoxic hepatitis

**DOI:** 10.1186/s13613-018-0356-z

**Published:** 2018-01-30

**Authors:** Astrid Van den broecke, Laura Van Coile, Alexander Decruyenaere, Kirsten Colpaert, Dominique Benoit, Hans Van Vlierberghe, Johan Decruyenaere

**Affiliations:** 1 Faculty of Medicine and Health Sciences, Ghent University, Ghent, Belgium; 2 Department of Internal Medicine, Ghent University Hospital, Ghent, Belgium; 3 Department of Intensive Care Medicine, Ghent University Hospital, De Pintelaan 185, 9000 Ghent, Belgium; 4 Department of Hepatology and Gastro-Enterology, Ghent University Hospital, Ghent, Belgium

**Keywords:** Critical care medicine, Critically ill, Epidemiology, Hypoxic hepatitis, Intensive care medicine, Ischemic hepatitis, Liver failure, Mortality, Outcome, Shock liver

## Abstract

**Background:**

Hypoxic hepatitis (HH) is a type of acute hepatic injury that is histologically characterized by centrilobular liver cell necrosis and that is caused by insufficient oxygen delivery to the hepatocytes. Typical for HH is the sudden and significant increase of aspartate aminotransferase (AST) in response to cardiac, circulatory or respiratory failure. The aim of this study is to investigate its epidemiology, causes, evolution and outcome.

**Methods:**

The screened population consisted of all adults admitted to the intensive care unit (ICU) at the Ghent University Hospital between January 1, 2007 and September 21, 2015. HH was defined as peak AST > 5 times the upper limit of normal (ULN) after exclusion of other causes of liver injury. Thirty-five variables were retrospectively collected and used in descriptive analysis, time series plots and Kaplan–Meier survival curves with multi-group log-rank tests.

**Results:**

HH was observed in 4.0% of the ICU admissions at our center. The study cohort comprised 1116 patients. Causes of HH were cardiac failure (49.1%), septic shock (29.8%), hypovolemic shock (9.4%), acute respiratory failure (6.4%), acute on chronic respiratory failure (3.3%), pulmonary embolism (1.4%) and hyperthermia (0.5%). The 28-day mortality associated with HH was 45.0%. Mortality rates differed significantly (*P* = 0.007) among the causes, ranging from 33.3% in the hyperthermia subgroup to 52.9 and 56.2% in the septic shock and pulmonary embolism subgroups, respectively. The magnitude of AST increase was also significantly correlated (*P* < 0.001) with mortality: 33.2, 44.4 and 55.4% for peak AST 5–10× ULN, 10–20× ULN and > 20× ULN, respectively.

**Conclusion:**

This study surpasses by far the largest cohort of critically ill patients with HH. HH is more common than previously thought with an ICU incidence of 4.0%, and it is associated with a high all-cause mortality of 45.0% at 28 days. The main causes of HH are cardiac failure and septic shock, which include more than 3/4 of all episodes. Clinicians should search actively for any underlying hemodynamic or respiratory instability even in patients with moderately increased AST levels.

## Background

Hypoxic hepatitis (HH), also referred to as “ischemic hepatitis” or “shock liver,” is a type of acute hepatic injury that is histologically characterized by centrilobular liver cell necrosis and that is caused by insufficient oxygen delivery to the hepatocytes [1]. Typical for this form of liver cell necrosis is the sudden and significant increase of aspartate aminotransferase (AST) in response to cardiac, circulatory or respiratory failure [1]. Although often missed, HH is a fairly common cause of hepatic dysfunction in an intensive care unit (ICU) with a pooled incidence of 2.5% from a recent meta-analysis of 1782 patients [2]. Its incidence varies widely among published studies, ranging from 0.16 to 12%, depending upon institution, population studied and definition used [1–8]. However, a high associated mortality of approximately 50% has been consistently observed in all studies [2–6, 8].

The major causes of HH are septic shock, respiratory failure and cardiogenic shock [1, 2]. Possible pathophysiological mechanisms include (1) ischemia due to reduced blood supply (forward failure) or due to right heart failure (backward failure) with venous congestion, (2) hypoxemia due to reduced blood oxygenation and (3) increased oxygen consumption due to elevated metabolic demand (e.g., in severe hyperthermia or septic shock) [1, 9]. Patients with comorbidities are more likely to develop HH, as they have an increased vulnerability even to minor hemodynamic or respiratory insults, such as short periods of hypotension or hypoxemia. These comorbidities contribute substantially to the high mortality associated with HH [4, 7, 8].

HH is reflected by a typical pattern of liver enzyme alterations. It presents with a sudden and significant increase of AST, alanine aminotransferase (ALT) and lactate dehydrogenase (LDH), reaching their peak levels around 24 h after ICU admission [4] and declining steadily to baseline within 10–15 days [1]. Initially, AST exceeds ALT, but as ALT declines more slowly, a reversal of the AST/ALT ratio is observed within 3 days after the peak [5, 6, 8, 10]. Although this biochemical pattern is highly suggestive of HH, it is not pathognomonic and warrants further evaluation [1]. Other common causes of significant increases in aminotransferase levels are drug-induced liver injury (e.g., acetaminophen toxicity) and acute viral hepatitis. However, studies have shown that a sudden and significant increase of AST is caused by HH in more than 50% of the cases [11, 12]. Furthermore, HH is frequently associated with a prolonged prothrombin time and accompanied by additional evidence of end-organ hypoperfusion, such as impaired renal function and increased lactate level [3–6]. A rapid rise and subsequent fall in aminotransferase levels with reversal of the initial AST/ALT ratio, a prolongation of prothrombin time and an increase in serum creatinine level comprise a triad of biochemical abnormalities that can suggest the diagnosis of HH, as proposed by Raurich et al. [6].

Currently, only limited data from small retrospective studies are available, making HH an understudied disease. The largest cohort described to date was recently published and included 565 patients [8]. The aim of this study is to investigate in detail the epidemiology, causes, evolution and outcome of HH in a large single-center cohort. More insight in HH may improve awareness and facilitate earlier diagnosis.

## Methods

### Study cohort and data collection

**Fig. 1:**
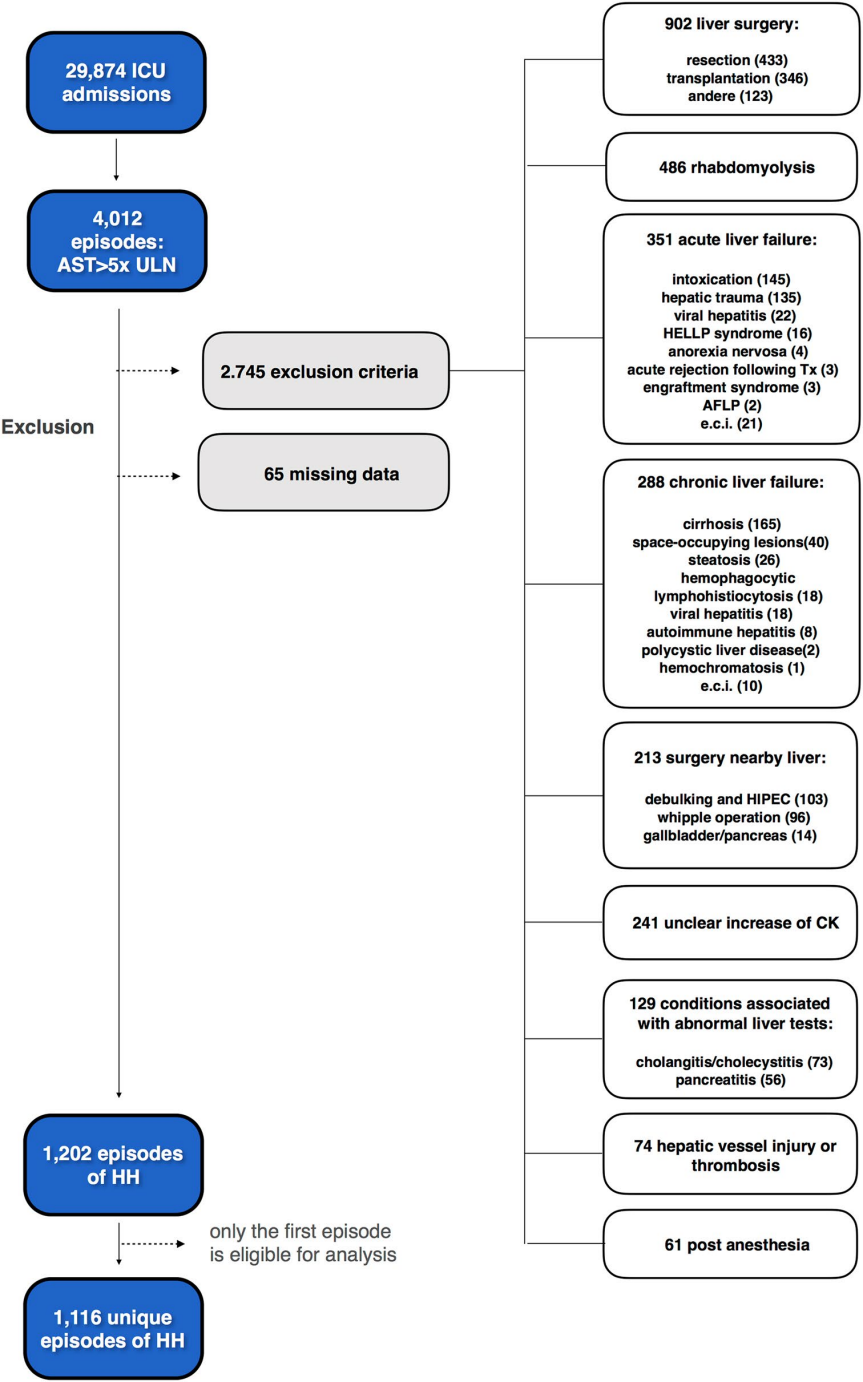
Flow diagram of the exclusion criteria. *AFLP* acute fatty liver of pregnancy, *AST* aspartate aminotransferase, *CK* creatine kinase, *e.c.i* e causa ignota, *HELLP* hemolysis, elevated liver enzymes and low platelet count, *HH* hypoxic hepatitis, *HIPEC* hyperthermic intraperitioneal chemotherapy, *ICU* intensive care unit

The screened population consisted of all consecutive adults (≥ 18 years, *n* = 29,874) who were admitted to the surgical, cardiac or medical ICU at the Ghent University Hospital between January 1, 2007 and September 21, 2015. HH was defined as a significant but transient increase in AST level above 5 times the upper limit of normal (ULN) after exclusion of other potential causes of liver injury. Different AST cutoff values for defining HH are used in the literature [2]. As HH has been histologically proven to occur even in patients with moderately elevated AST levels (at AST levels of 252 and 300 IU/L in the cohort of Cohen et al. [13] and Bynum et al. [14], respectively), a cutoff of at least 5 times the ULN was used in this study, i.e., 155 and 185 U/L for females and males, respectively. The 4012 identified patients whose AST level exceeded our cutoff value were evaluated for the presence of HH by three independent experts based on the pattern of liver enzyme alterations, the daily clinical notes and the discharge summaries. The sine qua non was the exposure to a hemodynamic or respiratory insult preceding the AST increase and the exclusion of other potential causes of liver injury. A flow diagram of the exclusion criteria is shown in Fig. 1. Reasons for exclusion were (1) acute liver failure, (2) chronic liver failure, (3) other conditions associated with abnormal liver tests such as cholangitis and pancreatitis, (4) liver surgery, (5) surgery near the liver, (6) hepatic vessel injury or thrombosis, (7) rhabdomyolysis, (8) an unclear increase of creatine kinase (CK), (9) post-anesthesia without overt evidence of an acute cardiac or respiratory event perioperatively, (10) missing data and (11) duplicate patients. (In patients having developed multiple episodes of HH during the study period, only the first episode is eligible for analysis.) Rhabdomyolysis was defined as serum CK exceeding 5 times the ULN (i.e., 850 and 974 U/L for females and males, respectively) with a CK-MB/CK ratio below 6% [6]. Patients with elevated CK levels but with unknown CK-MB value were excluded due to the uncertain CK increase. This study was approved by the Ethics Committee of the Ghent University Hospital (project numbers 2015/0796-0797). Due to the retrospective nature of this study, the need for informed consent was waived.

Routinely available biochemical parameters were recorded, including AST (U/L), ALT (U/L), LDH (U/L), bilirubin (mg/dL), alkaline phosphatase (AP) (U/L), gamma-glutamyl transpeptidase (U/L), lipase (U/L), international normalized ratio (INR), platelet count (10^3^/µL), white blood cell count (10^3^/µL), hemoglobin (g/dL), CK (U/L), creatinine (mg/dL), urea (mg/dL) and lactate (mg/dL). Values at specific time points were estimated using linear interpolation between consecutive recorded values. Parameters related to patient characteristics included sex, age, body mass index (BMI) and comorbidities (diabetes mellitus, cardiac function and chronic respiratory disease). Parameters related to the episode of HH included cause, severity of illness scores [acute physiology II score (APS-II) and the simplified acute physiology II score (SAPS-II)], supportive therapy (inotropic agents, vasopressor agents, mechanical ventilation, intra-aortic balloon pump (IABP) and need for dialysis), ICU and hospital length of stay, duration of the HH episode, and ICU and in-hospital mortality at 28 days. The peak of HH was defined as the point in time (T-ASTmax) when AST reached its peak value (ASTmax). In this study, the time origin was set to T-ASTmax (designated as time 0) and all recorded values are expressed in time relative to T-ASTmax for optimal comparison. Severity of illness scores were recorded over a time span of 24 h around T-ASTmax. Three categories for severity of HH were used: “5–10× ULN”, “10–20× ULN” and “> 20× ULN”. Seven underlying causes of HH were defined: cardiac failure, septic shock, hypovolemic shock, pulmonary embolism, acute respiratory failure, acute on chronic respiratory failure and hyperthermia.

### Statistical analysis

Categorical data are reported as counts and percentages. Continuous data are reported as the median with the first (Q1) and third (Q3) quartiles. For categorical variables, comparisons between groups are performed using the Pearson’s Chi square test for contingency. For continuous variables, a permutation test based on difference in medians between groups is used. A Tukey-like approach with permutation resampling is applied to adjust *P* values for multiple pairwise comparisons. All statistical tests are performed as two-sided tests at a significance level of 0.05. The time trend of laboratory variables is graphically assessed using time series plots and stacked bar charts. Kaplan–Meier survival curves until 28 days after T-ASTmax and multi-group log-rank tests are used to compare the all-cause mortality between groups. Statistical analysis is performed using R version 3.3.2 [15].

## Results

### Patient and episode characteristics

During the study period, 29,874 adult patients with a male-to-female ratio of approximately 3:2 were admitted to the ICU at our center, of whom 4012 patients had peak AST levels exceeding 5 times the ULN. In 30.0% (1202/4012) of the cases, the elevation of AST was caused by HH, resulting in an overall ICU incidence of 4.0% (1202/29,874). As only the first episode was eligible for analysis in patients with multiple episodes of HH during the study period, the final study cohort comprised 1116 patients.

**Fig. 2:**
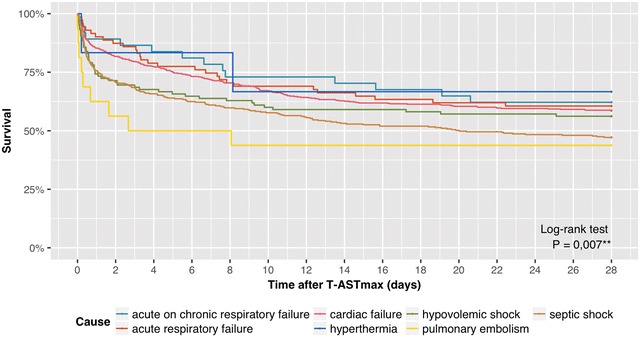
Survival curves by cause of hypoxic hepatitis. T-ASTmax is designated as the time 0. *T-ASTmax* time point of maximum AST value

The all-cause mortality of patients admitted to the ICU at our center during the study period was 7.7% (2302/29,874). Among these non-survivors, 19.8% (455/2302) had developed HH during their ICU stay. The all-cause mortality associated with HH was 45.0% (502/1116) at 28 days, of which 90.6% occurred during ICU stay. The survival curves by cause of HH are presented in Fig. 2.

In our study cohort, the median age was 66.0 (Q1–Q3 55.0–74.0) years with a male-to-female ratio of 3:2. The median SAPS-II score at T-ASTmax was 66.0 (Q1–Q3 43.0–81.0). 74.9% of patients required mechanical ventilation. Vasopressor and inotropic agents were used in 60.8 and 43.2%, respectively, and 9.5% were on dialysis at T-ASTmax. The causes of HH, in decreasing order of frequency, were cardiac failure (49.1%), septic shock (29.8%), hypovolemic shock (9.4%), acute respiratory failure (6.4%), acute on chronic respiratory failure (3.3%), pulmonary embolism (1.4%) and hyperthermia (0.5%). An episode of HH (length of time that AST levels are exceeding 5 times the ULN) had a median duration of 54.3 (Q1–Q3 26.4–94.2) h, during which the median recovery time (duration from peak AST to levels below 5 times the ULN) was 34.7 (Q1–Q3 14.6–67.2) h.

Survivors had significantly lower severity of illness scores (median SAPS-II score of 54.0 vs. 76.0, *P* < 0.001 and median APS-II score 25.0 vs. 30.0, *P* = 0.005) as compared to non-survivors. They were less likely to have septic shock (25.6 vs. 35.1%, *P* = 0.003). Peak AST levels above 20 times ULN were less commonly seen in survivors (33.4 vs. 50.8%, *P* < 0.001). More characteristics, classified according to survival status, are presented in Table 1.

**Table 1:** Characteristics of patients with HH classified by in-hospital 28-day mortality

Variable	Patients				Variable	Patients			
	Total (*n* = 1116)	In-hospital 28-day mortality		*P*		Total (*n* = 1116)	In-hospital 28-day mortality		*P*
		No (*n* = 614)	Yes (*n* = 502)				No (*n* = 614)	Yes (*n* = 502)	
Sex^a^					ASTmax^a^				
Male	61.3% (684)	62.2% (382)	60.2% (302)	0.483	5–10× ULN	35.4% (395)	43.0% (264)	26.1% (131)	*<* *0.001*
Age^b^ (year)	66.0 (55.0–74.0)	63.0 (53.0–73.0)	68.0 (58.0–76.8)	*<* *0.001*	10–20× ULN	23.4% (261)	23.6% (145)	23.1% (116)	0.985
BMI^b^ (kg/m^2^)	25.4 (22.9–28.9)	25.4 (23.0–28.5)	25.4 (22.9–29.4)	0.774	> 20× ULN	41.2% (460)	33.4% (205)	50.8% (255)	*<* *0.001*
Cause^a^					Supportive therapy^c^				
Cardiac failure	49.1% (548)	52.3% (321)	45.2% (227)	0.103	Ventilation^a^	74.9% (836)	66.4% (408)	85.3% (428)	*<* *0.001*
Septic shock	29.8% (333)	25.6% (157)	35.1% (176)	*0.003*	Medication				
Hypovolemic shock	9.4% (105)	9.6% (59)	9.2% (46)	0.999	Inotropic agents^a^	43.2% (482)	39.3% (241)	48.0% (241)	*0.003*
Acute respiratory failure	6.4% (71)	7.0% (43)	5.6% (28)	0.927	Vasopressor agents^a^	60.8% (678)	51.1% (314)	72.5% (364)	*<* *0.001*
Acute on chronic respiratory failure	3.3% (37)	3.7% (23)	2.8% (14)	0.956	Dialysis^a^	9.5% (106)	6.8% (42)	12.7% (64)	*<* *0.001*
Pulmonary embolism	1.4% (16)	1.1% (7)	1.8% (9)	0.945	IABP^a^	17.5% (195)	19.5% (120)	14.9% (75)	*0.044*
Hyperthermia	0.5% (6)	0.7% (4)	0.4% (2)	0.995	LOS				
Comorbidities					ICU^b^ (day)	4.4 (1.2–11.7)	6.4 (2.7–17.7)	1.8 (0.4–7.1)	*<* *0.001*
Echocardio^a^					Hospital^b^ (day)	12.1 (3.0–28.9)	25.7 (13.9–48.1)	2.1 (0.4–8.1)	*<* *0.001*
Overall				0.406	Duration				
Normal	40.0% (446)	42.0% (258)	37.5% (188)	–	Episode of HH^b^ (h)	54.3 (26.4–94.2)	53.4 (23.9–94.2)	56.7 (30.7–94.1)	0.527
LV dysfunction	25.1% (280)	23.5% (144)	27.1% (136)	–	Recovery of HH^b^ (h)	34.7 (14.6–67.2)	35.5 (14.1–68.0)	32.6 (16.2–61.4)	0.393
Missing	22.2% (248)	22.5% (151)	23.3% (117)	–	28-day mortality				
LV and RV dysfunction	8.0% (89)	8.0% (49)	8.0% (40)	–	ICU^a^	40.8% (455)	0.0% (0)	90.6% (455)	*<* *0.001*
RV dysfunction	4.7% (53)	5.2% (32)	4.2% (21)	–	In-hospital^a^	45.0% (502)	0.0% (0)	100.0% (502)	*<* *0.001*
DM^a^	18.7% (209)	17.4% (107)	20.3% (102)	0.218					
Chronic respiratory failure^a^	11.4% (127)	10.7% (66)	12.2% (61)	0.287					
SOI scores^c^									
APS-II^b^	28.0 (21.0–34.0)	25.0 (18.0–32.0)	30.0 (24.0–35.2)	*0.005*					
SAPS-II^b^	66.0 (46.0–81.0)	54.0 (36.0–71.0)	76.0 (58.0–89.0)	*<* *0.001*					

*APS-II* acute physiology II, *AST* aspartate aminotransferase, *ASTmax* maximum AST value, *BMI* body mass index, *HH* hypoxic hepatitis, *IABP* intra-aortic balloon pump, *ICU* intensive care unit, *LOS* length of stay, *LV* left ventricle, *P P* value, *Q1* first quartile, *Q3* third quartile, *RV* right ventricle, *SAPS-II* simplified acute physiology II, *SOI* severity of illness, *T-ASTmax* time point of maximum AST value, *ULN* upper limit of normal

Italics indicate the significant *P* values

^a^% (*n*)

^b^Median (Q1–Q3)

^c^At T-ASTmax ± 12 h

The three ASTmax subgroups (“5–10× ULN” vs. “10–20× ULN” vs. “> 20× ULN”) were also compared with each other. The causes of HH were equally represented within each subgroup. Patients with higher peak AST levels had higher severity of illness scores (median SAPS-II score of 57.0 vs. 60.0 vs. 73.0, *P* < 0.001; median APS-II score of 24.0 vs. 26.5 vs. 32.0, *P* < 0.001) and a higher need for supportive therapy, such as inotropic agents (38.5 vs. 37.9 vs. 50.2%, *P* < 0.001), vasopressor agents (50.9 vs. 59.4 vs. 70.0%, *P* < 0.001), dialysis (4.8 vs. 8.4 vs. 14.1%, *P* < 0.001) and IABP (13.7 vs. 20.7% vs. 18.9%, *P* = 0.041). In addition, higher peak AST levels were associated with higher 28-day mortality rates (33.2 vs. 44.4 vs. 55.4%, *P* < 0.001). More characteristics, classified according to severity of HH and to cause of HH, are presented in Tables 2 and 3, respectively.

**Table 2:** Characteristics of patients with HH classified by ASTmax subgroup

Variable	Patients				Variable	Patients			
	ASTmax subgroup			*P*		ASTmax subgroup			*P*
	5× ULN–10× ULN (*n* = 395)	10× ULN–20× ULN (*n* = 261)	> 20× ULN (*n* = 460)			5× ULN–10× ULN (*n* = 395)	10× ULN–20× ULN (*n* = 261)	> 20× ULN (*n* = 460)	
Sex^a^					SOI scores^c^				
Male	60.5% (239)	62.5% (163)	61.3% (282)	0.882	APS-II^b^	24.0 (18.0–31.0)	26.5 (20.0–32.2)	32.0 (24.0–37.9)	*<* *0.001*
Age^b^ (year)	63.0 (45.0–73.5)	66.0 (56.0–75.0)	67.0 (55.8–75.0)	*0.014*	SAPS-II^b^	57.0 (36.2–74.0)	60.0 (41.0–78.2)	73.0 (56.0–86.5)	*<* *0.001*
BMI^b^ (kg/m^2^)	25.5 (23.1–28.7)	25.5 (22.9–29.2)	25.1 (22.9–28.9)	0.647	Supportive therapy^c^				
Cause^a^					Ventilation^a^	71.9% (284)	72.4% (189)	78.9% (363)	*0.035*
Overall				0.135	Medication				
Cardiac failure	50.6% (200)	49.8% (130)	47.4% (218)	–	Inotropic agents^a^	38.5% (152)	37.9% (99)	50.2% (231)	*<* *0.001*
Septic shock	29.4% (116)	26.1% (68)	32.4% (149)	–	Vasopressor agents^a^	50.9% (201)	59.4% (155)	70.0% (322)	*<* *0.001*
Hypovolemic shock	7.6% (30)	10.3% (27)	10.4% (48)	–	Dialysis^a^	4.8% (19)	8.4% (22)	14.1% (65)	*<* *0.001*
Acute respiratory failure	7.1% (28)	9.2% (24)	4.1% (19)	–	IABP^a^	13.7% (54)	20.7% (54)	18.9% (87)	*0.041*
Acute on chronic respiratory failure	3.3% (13)	1.9% (5)	4.1% (19)	–	LOS				
Pulmonary embolism	1.5% (6)	1.5% (4)	1.3% (6)	–	ICU^b^ (day)	4.6 (1.6–11.2)	4.8 (1.4–11.2)	4.0 (0.7–12.4)	0.325
Hyperthermia	0.5% (2)	1.1% (3)	0.2% (1)	–	Hospital^b^ (day)	12.5 (6.5–29.5)	13.1 (4.1–27.6)	10.2 (0.9–29.8)	0.250
Comorbidities					Duration				
Echocardio^a^					Episode of HH^b^ (h)	23.6 (11.4–37.2)	56.8 (40.5–79.6)	104.7 (78.1–132.0)	*<* *0.001*
Normal	43.8% (173)	34.5% (90)	39.8% (183)	0.234	Recovery of HH^b^ (h)	12.6 (6.1–22.2)	36.2 (22.7–52.0)	77.7 (56.6–102.4)	*<* *0.001*
LV dysfunction	20.5% (81)	27.6% (72)	27.1% (136)	0.136	28-day mortality				
Missing	25.3% (100)	26.8% (70)	17.0% (78)	*0.008*	ICU^a^	29.4% (116)	38.3% (100)	52.0% (239)	*<* *0.001*
LV and RV dysfunction	5.8% (23)	8.4% (22)	9.6% (44)	0.452	In-hospital^a^	33.2% (131)	44.4% (116)	55.4% (255)	*<* *0.001*
RV dysfunction	4.6% (18)	2.7% (7)	6.1% (28)	0.422					
DM^a^	14.9% (59)	21.1% (55)	20.7% (95)	0.055					
Chronic respiratory failure^a^	12.2% (48)	10.7% (28)	11.1% (51)	0.826					

*APS-II* acute physiology II, *AST* aspartate aminotransferase, *ASTmax* maximum AST value, *BMI* body mass index, *HH* hypoxic hepatitis, *IABP* intra-aortic balloon pump, *ICU* intensive care unit, *LOS* length of stay, *LV* left ventricle, *P P* value, *Q1* first quartile, *Q3* third quartile, *RV* right ventricle, *SAPS-II* simplified acute physiology II, *SOI* severity of illness, *T-ASTmax* time point of maximum AST value, *ULN* upper limit of normal

Italics indicate the significant *P* values

^a^% (*n*)

^b^Median (Q1–Q3)

^c^At T-ASTmax ± 12 h

**Table 3:** Characteristics of patients with HH classified by clinical cause

Variable	Cause							
	Cardiac failure (*n* = 548)	Septic shock (*n* = 333)	Hypovolemic shock (*n* = 105)	Acute respiratory failure (*n* = 71)	Acute on chronic respiratory failure (*n* = 37)	Pulmonary embolism (*n* = 16)	Hyperthermia (*n* = 6)	*P*
Sex^a^								
Male	63.1% (346)	61.6 (205)	57.1% (60)	54.9% (39)	54.1% (20)	62.5 (10)	66.7% (4)	0.721
Age^b^ (year)	68.0 (58.0–76.0)	64.0 (52.0–74.0)	64.0 (56.0–73.0)	59.0 (48.5–71.5)	65.0 (61.0–72.0)	57.5 (47.5–61.5)	54.5 (35.8–64.2)	*0.003*
BMI^b^ (kg/m^2^)	25.5 (23.3–28.7)	25.0 (22.1–29.3)	26.2 (23.4–28.9)	24.2 (22.0–27.8)	24.6 (22.1–27.3)	27.8 (25.1–31.0)	27.6 (23.7–29.3)	0.202
Comorbidities								
Echocardio^a^								
Normal	32.7% (179)	51.1% (170)	43.8% (46)	36.6% (26)	48.6% (18)	18.8% (3)	66.7% (4)	*<* *0.001*
LV dysf.	36.5% (200)	15.9% (53)	5.7% (6)	21.1% (15)	10.8% (4)	6.2% (1)	16.7% (1)	*<* *0.001*
Missing	13.3% (73)	26.1% (87)	46.7% (49)	35.2% (25)	24.3% (9)	25.0% (4)	16.7% (1)	*<* *0.001*
LV and RV dysf.	13.0% (71)	3.3% (11)	1.0% (1)	4.2% (3)	2.7% (1)	12.5% (2)	0.0% (0)	*<* *0.001*
RV dysf.	4.6% (25)	3.6% (12)	2.9% (3)	2.8% (2)	13.5% (5)	37.5% (6)	0.0% (0	*<* *0.001*
DM^a^	20.8% (114)	19.2% (64)	10.5% (11)	12.7% (9)	24.3% (9)	12.5% (2)	0.0% (0)	0.103
Chronic respiratory failure^a^	8.4% (46)	9.6% (32)	9.5% (10)	0.0% (%)	100.0% (37)	12.5% (2)	0.0% (0)	*<* *0.001*
SOI scores^c^								
APS-II^b^	28.0 (23.0–35.0)	26.0 (21.0–35.0)	26.0 (18.5–32.5)	30.0 (28.0–34.0)	26.5 (24.2–30.8)	36.5 (36.2–36.8)	21.5 (19.2–25.0)	0.590
SAPS-II^b^	66.0 (44.0–79.0)	67.0 (49.0–84.0)	65.0 (48.0–82.0)	63.0 (31.0–79.0)	67.5 (55.0–73.5)	83.0 (69.2–99.2)	41.5 (34.0–48.2)	0.171
ASTmax^a^								
Overall								0.135
5–10× ULN	36.5% (200)	34.8% (116)	28.6% (30)	39.4% (28)	35.1% (13)	37.5% (6)	33.3% (2)	–
10–20× ULN	23.7% (130)	20.4% (68)	25.7% (27)	33.8% (24)	13.5% (5)	25.0% (4)	50.0% (3)	–
> 20× ULN	39.8% (218)	44.7% (149)	45.7% (48)	26.8% (19)	51.4% (19)	37.5% (6)	16.7% (1)	–
Supportive therapy^c^								
Ventilation^a^	79.2% (434)	73.0% (243)	70.5% (74)	60.6% (43)	64.9 (24)	75.0% (12)	100.0% (6)	*0.004*
Medication								
Inotropic agents^a^	63.1% (346)	26.4% (88)	21.0% (22)	12.7% (9)	21.6% (8)	43.8% (7)	33.3% (2)	*<* *0.001*
Vasopressor agents^a^	58.6% (321)	69.1% (230)^d^	61.9% (65)	46.5% (33)	43.2% (16)	75.0% (12)	16.7% (1)	*<* *0.001*
Dialysis^a^	10.4% (57)	11.1% (37)	2.9% (3)	9.9% (7)	2.7% (1)	0.0% (0)	16.7% (1)	0.090
IABP^a^	32.8% (180)	4.2% (14)	0.0% (0)	1.4% (1)	0.0% (0)	0.0% (0)	0.0% (0)	*<* *0.001*
LOS								
ICU^b^ (day)	4.6 (1.4–10.5)	4.7 (0.9–15.8)	2.6 (0.7–7.8)	6.3 (2.3–15.0)	3.2 (1.4–7.8)	2.2 (0.3–4.1)	7.7 (6.6–14.2)	0.135
Hospital^b^ (day)	11.1 (4.6–25.7)	12.3 (1.1–37.9)	11.2 (0.9–28.8)	14.3 (5.2–30.2)	20.6 (7.6–27.7)	5.4 (0.3–19.4)	43.4 (10.2–97.4)	*0.037*
Duration								
Episode of HH^b^ (h)	50.6 (24.9–99.3)	54.5 (25.1–87.2)	69.4 (36.8–89.5)	71.3 (39.0–90.0)	28.4 (15.1–77.2)	69.5 (28.0–139.0)	54.6 (35.4–74.0)	0.137
Recovery of HH^b^ (h)	33.8 (3.4–67.5)	34.3 (14.3–60.9)	41.7 (21.1–65.1)	31.0 (16.2–62.1)	39.9 (11.6–89.3)	35.3 (10.5–65.4)	29.6 (21.7–43.3)	0.810
28-day mortality								
ICU^a^	37.8% (207)	48.3% (161)	40.0% (42)	33.8% (24)	27.0% (10)	56.2% (9)	33.3% (2)	*0.012*
In-hospital^a^	41.4% (227)	52.9% (176)	43.8% (46)	39.4% (28)	37.8% (14)	56.2% (9)	33.3% (2)	*0.029*

*APS-II* acute physiology II, *AST* aspartate aminotransferase, *ASTmax* maximum AST value, *BMI* body mass index, *HH* hypoxic hepatitis, *IABP* intra-aortic balloon pump, *ICU* intensive care unit, *LOS* length of stay, *LV* left ventricle, *P P* value, *Q1* first quartile, *Q3* third quartile, *RV* right ventricle, *SAPS-II* simplified acute physiology II, *SOI* severity of illness, *T-ASTmax* time point of maximum AST value, *ULN* upper limit of normal

^a^% (*n*)

^b^Median (Q1–Q3)

^c^At T-ASTmax ± 12 h

^d^A condition for septic shock was the need of vasopressor agents. In this table, the proportion of patients receiving vasopressor agents only at T-ASTmax is given

### Trend of liver tests

**Fig. 3:**
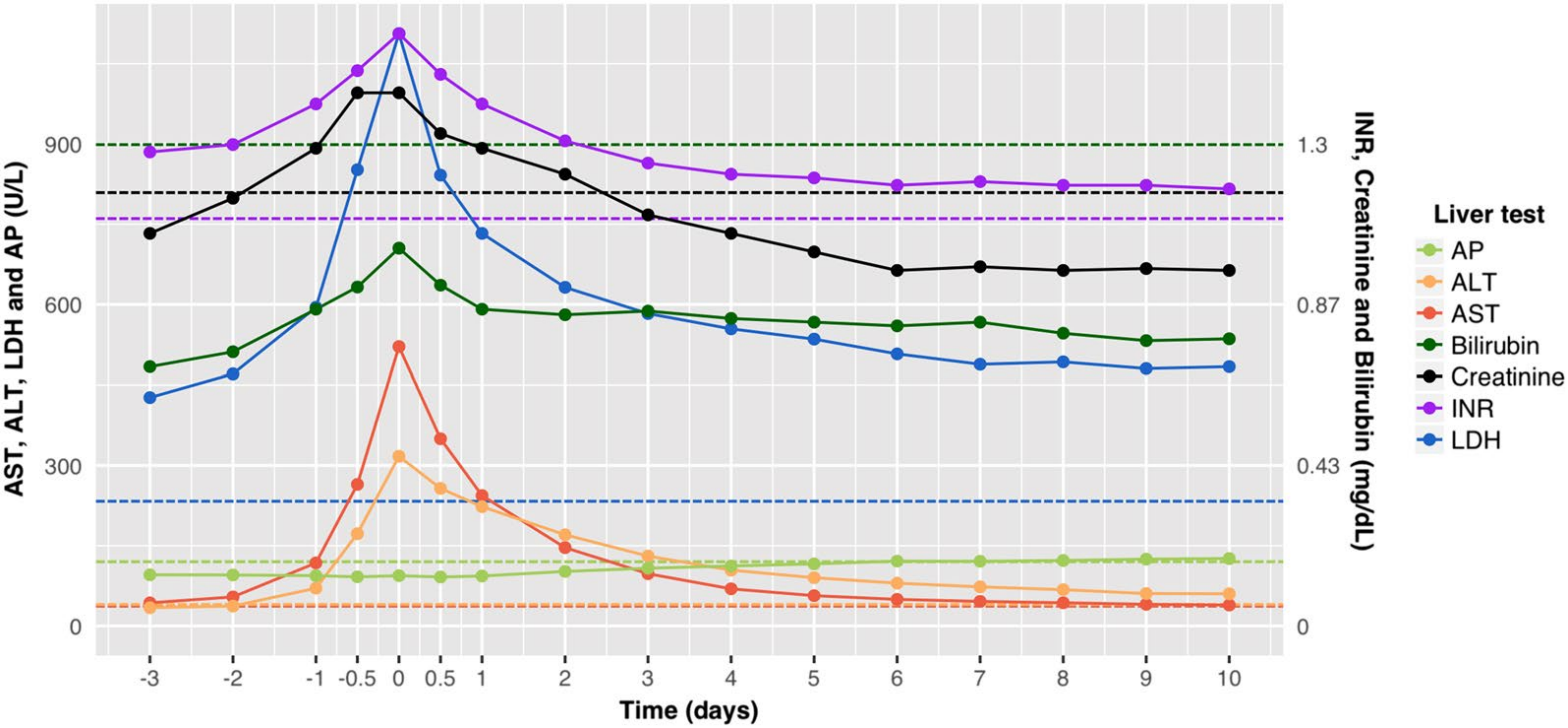
Time trend of liver tests in hypoxic hepatitis. Time trend of the median AP, ALT, AST, bilirubin, creatinine, INR and LDH levels in hypoxic hepatitis. T-ASTmax is designated as time 0. AST, ALT, LDH and AP levels are measured in U/L and are plotted using the left *y*-axis. Bilirubin and creatinine levels are measured in mg/dL and plotted using the right y-axis. INR has no unit, but is also plotted using the right *y*-axis. *AP* alkaline phosphatase, *ALT* alanine aminotransferase, *AST* aspartate aminotransferase, *INR* international normalized ratio, *LDH* lactate dehydrogenase

The time trend of the liver tests is illustrated in Fig. 3. Once the AST level exceeded 5 times the ULN (designated as start of HH), it reached a median peak level of 521.2 (Q1–Q3 269.1–1581.6) U/L within a median of 16.3 (Q1–Q3 7.9–26.8) h. Subsequently, AST started to decline, dropping below 5 times the ULN after a median time of 34.7 (Q1–Q3 14.6–67.2) h and normalizing after a median time of 6.4 (Q1–Q3 3.9–10.9) days in survivors.

ALT and LDH peaked at median values of 332.0 (Q1–Q3 149.4–953.6)  U/L and 1180.6 (Q1–Q3 684.1–2770.8) U/L within a median time of 22.0 (Q1–Q3 9.7–43.0) and 21.4 (Q1–Q3 10.0–43.6) h after start of HH, respectively. In survivors, LDH returned to baseline (< 233 U/L) within a median of 5.1 (Q1–Q3 1.3–15.9) days after its peak. ALT declined more slowly, returning to baseline (< 31 and < 40 U/L for females and males, respectively) within 8.6 (Q1–Q3 3.9–16.5) days after its peak. When considering all patients, including those with AST already exceeding 5 times the ULN at ICU admission, ALT and LDH peaked at T-ASTmax in the majority of patients (65.5 and 53.1%, respectively) and to a lesser extent after T-ASTmax (26.2 and 29.8%, respectively).

The median relative increase of AST, ALT and LDH at T-ASTmax was 15.0, 8.7 and 4.7 times their ULN, respectively. LDH exceeded both AST and ALT at all time points in more than 80% of patients. The median AST/ALT ratio was 1.6 at the start of HH, increased to 1.8 at T-ASTmax and subsequently declined, reversing within a median of 32.5 h after T-ASTmax.

**Fig. 4:**
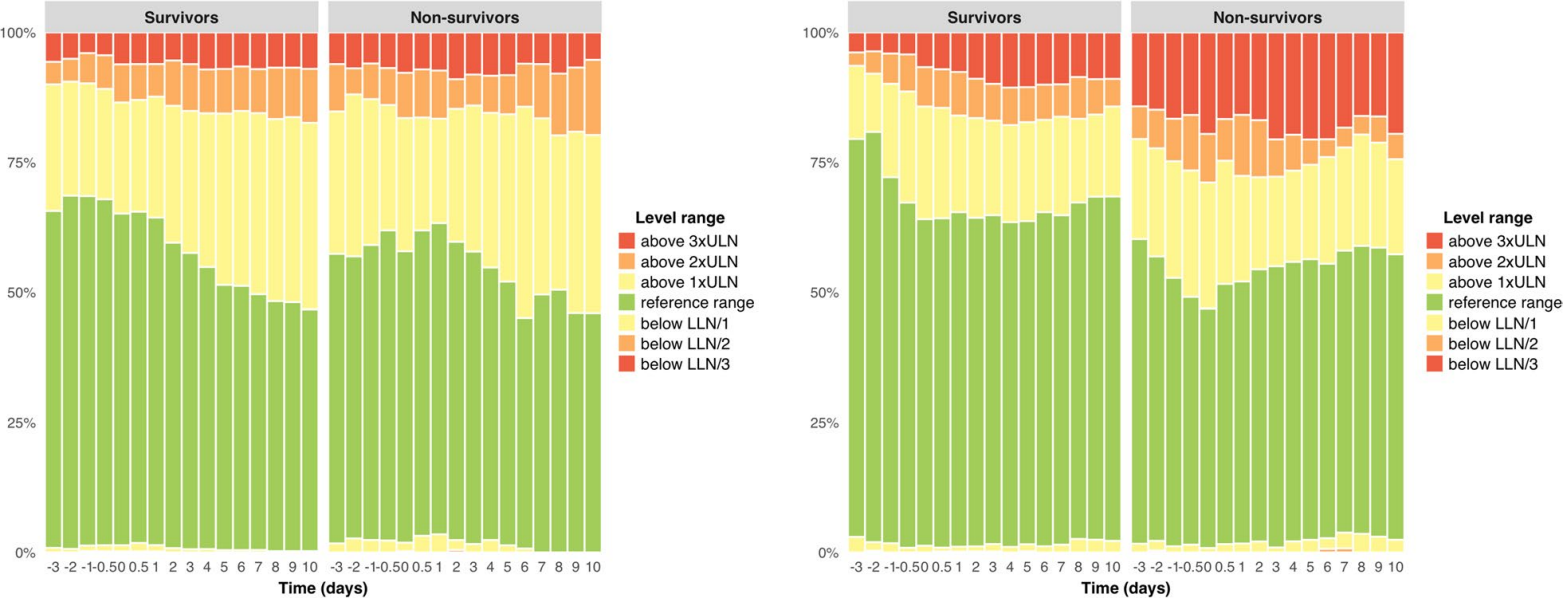
**a** AP level range over time; **b** bilirubin level range over time. Evolution of the proportion of survivors and non-survivors with alkaline phosphatase (left) and bilirubin (right) levels within and outside the reference range. T-ASTmax is designated as time 0. *AP* alkaline phosphatase, *LLN* lower limit of normal, *ULN* upper limit of normal

In addition, Fig. 3 illustrates that the median INR, creatinine and bilirubin levels also peaked at T-ASTmax, albeit to a much less extent, with median bilirubin level never exceeding the ULN. This is also illustrated in Fig. 4. Both in survivors and non-survivors, the proportion of patients with bilirubin level above the ULN increased mildly at T-ASTmax and then gradually decreased again. The proportion of survivors with bilirubin above 3 times the ULN was limited and increased slightly later during ICU stay. Likewise, AP remained stable during HH, except for a mild increase later during ICU stay, as illustrated in Figs. 3 and 4.

The pattern of liver enzyme alterations described above was found in all ASTmax subgroups (see Appendix Figs. 5, 6, 7). Median values of other laboratory tests at start of HH and at T-ASTmax are presented in Table 4.

**Table 4:** Median laboratory values (Q1–Q3) at different time stages

Variable	At start of HH	At T-ASTmax
AST (U/L)	185.0 (155.0–185.0)	521.2 (269.1–1581.6)
ALT (U/L)	120.8 (70.1–193.5)	317.0 (143.5–924.5)
LDH (U/L)	691.8 (465.8–1025.7)	1106.4 (624.8–2602.8)
Bilirubin (mg/dL)	0.8 (0.5–1.6)	1.0 (0.6–2.0)
GGT (U/L)	73.1 (36.2–160.4)	82.5 (41.7–167.0)
AP (U/L)	88.2 (61.8–150.9)	94.0 (62.9–167.0)
Lipase (U/L)	32.4 (18.9–58.9)	33.5 (18.5–66.4)
INR	1.4 (1.2–1.8)	1.6 (1.3–2.2)
PC (10^3^/µL)	165.7 (103.6–241.0)	146.0 (79.5–218.0)
WBC (10^3^/µL)	12.5 (8.8–17.6)	13.2 (9.0–18.5)
Hb (g/dL)	10.1 (8.8–12.0)	9.8 (8.6–11.5)
CK (U/L)	214.0 (77.2–747.2)	322.2 (105.1–1102.9)
Creatinine (mg/dL)	1.4 (0.9–2.1)	1.4 (0.9–2.2)
Urea (mg/dL)	57.1 (38.9–91.3)	61.6 (40.6–96.4)
Lactate (mg/dL)	31.3 (16.2–62.2)	27.4 (14.7–56.2)

*AP* alkaline phosphatase, *ALT* alanine aminotransferase, *AST* aspartate aminotransferase, *CK* creatine kinase, *GGT* gamma-glutamyl transpeptidase, *Hb* hemoglobin, *INR* international normalized ratio, *LDH* lactate dehydrogenase, *PC* platelet count, *WBC* white blood cell count

### 28-day mortality

HH was associated with high mortality rates, especially early in its course. 18.5% died within 24 h after T-ASTmax. After this first day, the hazard declined dramatically, but remained quite high, resulting in 40.2 and 45.0% all-cause mortality at 14 and 28 days, respectively. 90.6% of deaths occurred during ICU stay.

Twenty-eight-day mortality rates differed significantly among the causes of HH (*P* = 0.007). The high number of early deaths in the pulmonary embolism, septic shock and hypovolemic shock subgroups mainly accounted for the early mortality associated with HH. 37.5, 24.3, 25.7% of these patients died within 24 h after T-ASTmax. The mortality rate in the pulmonary embolism subgroup exceeded at all times those of other subgroups and ultimately resulted in 56.2% mortality at 28 days. The septic shock and hypovolemic shock subgroups had a 28-day mortality of 52.9 and 43.8%, respectively. While the early mortality in the cardiac failure subgroup was less pronounced, yet still considerable as 14.8% of these patients died within 24 h, its 28-day mortality of 41.4% ranked fourth among all subgroups. The acute respiratory failure and acute on chronic respiratory failure subgroups had similar survival curves and outcome, with 39.4 and 37.8% mortality at 28 days. The lowest mortality rate of 33.3% at 28 days was observed in the hyperthermia subgroup. The survival curves by cause of HH are presented in Fig. 2.

The 28-day mortality also increased significantly (*P* < 0.001) with severity of HH, ranging from 33.2% in the “5–10× ULN” subgroup to 55.4% in the “> 20× ULN” subgroup. There was no significant difference (*P* = 0.363) in 28-day mortality between men (39.8%) and women (42.4%).

## Discussion

Hypoxic hepatitis is a type of acute hepatic injury in critically ill patients caused by cardiac, circulatory or respiratory failure. While commonly known and referred to as “shock liver” in daily clinical practice, little research has been done. However, early recognition of HH and management of its underlying cause and complications may improve ultimate outcome. For a long time, only studies with a relatively small number of patients have been conducted. In 2015, a meta-analysis of 24 studies that included 1782 patients summarized the available evidence, but still lacked detailed information on important patient characteristics, biochemical findings and clinical course [2]. To overcome these shortcomings, Aboelsoud et al. recently performed an extensive analysis of the Medical Information Mart for Intensive Care III (MIMIC-III) research database. Their study comprised 565 patients with HH and was the largest cohort study published to date [8].

In our study of 1116 critically ill patients with HH, we investigated in detail its incidence, causes, evolution and outcome. We used an AST cutoff of at least 5 times the ULN (i.e., 155 and 185 U/L for females and males, respectively) to define HH, while other studies have used higher cutoff values ranging from 400 to 3000 U/L [2]. HH has been histologically proven to occur even in patients with moderately elevated AST levels (at AST levels of 252 and 300 IU/L in the cohort of Cohen et al. [13] and Bynum et al. [14], respectively). Additionally, by using a lower cutoff, the sensitivity to identify patients with HH could be increased. However, this approach could also result in a lower specificity, leading to an increased number of patients that are falsely diagnosed as having HH. In order to maintain a high specificity, we have thoroughly reviewed the clinical notes, the time trend of liver tests and the discharge summaries of the 4012 identified patients whose AST level exceeded our cutoff value. Our high number of excluded patients (70.0%) after applying extensive exclusion criteria may reflect a high specificity. In contrast, Aboelsoud et al. [8] used a cutoff of 800 U/L for both AST and ALT to include patients, but only excluded 24.3% of these patients based on evidence of acetaminophen poisoning, acute viral hepatitis or liver surgery.

Our study uncovered interesting results. Firstly, it indicated that HH may be more common than previously thought. In our study, an incidence of 4.0% was observed, which is higher than the pooled incidence of 2.5% from a meta-analysis of 1782 patients [2] and the incidence of 1.5% in the MIMIC-III cohort [8]. Moreover, approximately one in five critically ill patients that have died during their ICU stay had developed HH. This higher incidence observed in our study is of course a direct consequence of the lower AST cutoff used. However, it should be noted that the underlying conditions, the typical liver test pattern and the considerable mortality of 33.2% at 28 days in patients with a less pronounced rise of AST (peak AST level between 5 and 10 times the ULN) are just as much compatible with the diagnosis of HH as in patients with higher peak AST level. Any sudden increase in aminotransferases above 5 times the ULN should therefore prompt the clinician to actively search for any changes in physiological parameters and maintain strict hemodynamic and respiratory control.

Secondly, the typical liver test pattern that suggests diagnosis of HH was observed in all subgroups of our study cohort. HH was characterized by (1) a sudden and significant increase of aminotransferases with an AST/ALT ratio greater than 1, (2) a similar rise in LDH mostly exceeding both AST and ALT and (3) a steady decline of aminotransferases with reversal of the AST/ALT ratio approximately 1.5 days after T-ASTmax. In survivors, LDH normalized first, followed by AST and finally ALT (median of 5.1, 6.4 and 8.6 days after T-ASTmax, respectively). Bilirubin followed a similar pattern as the aminotransferases, but the magnitude of its increase was much less pronounced, with median levels never exceeding the ULN. The associated prolongation of INR and impairment of renal function as described by Raurich et al. [6] were also confirmed in this study.

Finally, we observed a 28-day mortality of 45.0%, which is in line with the reported mortality of 49% from a meta-analysis of 1782 patients [2] and the mortality of 44.1% in the MIMIC-III cohort [8]. HH was especially associated with high mortality rates early in its course, with roughly 40% of deaths occurring within 24 h after T-ASTmax. This was mainly driven by the severity of the underlying causes [2], although the ultimate cause of death was not inferred in this study. The magnitude of AST increase also appeared to be correlated with mortality on univariate analysis.

Our study has a number of strengths. To our knowledge, it is by far the largest cohort study of patients with HH (*n* = 1116). It comprises twice as many patients as the second largest cohort study, which was recently published and included 565 patients [8]. Additionally, by using a low AST cutoff of 5 times the ULN while simultaneously excluding many conditions that could mimic HH, we are confident that we could identify nearly all patients with HH at our center with a low risk of misdiagnosis. Our study may have some limitations inherent in its retrospective design. However, we believe that the retrospective design had little to no impact on our results given the entirely computerized monitoring and management at our ICU, the detailed review of patient records and the high number of patients included. Nevertheless, we should be careful to generalize these results to a broader patient population since only critically ill patients older than 18 years and only from a single university hospital ICU were included.

## Conclusions

This study surpasses by far the largest cohort of critically ill patients with hypoxic hepatitis. HH is more common than previously though**t** with an ICU incidence of 4.0%, and it is associated with a high all-cause mortality of 45.0% at 28 days. The main causes of HH are cardiac failure and septic shock, which include more than 3/4 of all episodes. Clinicians should search actively for any underlying hemodynamic or respiratory instability even in patients with moderately increased AST levels.

## Figures and Tables

**Fig. 5:**
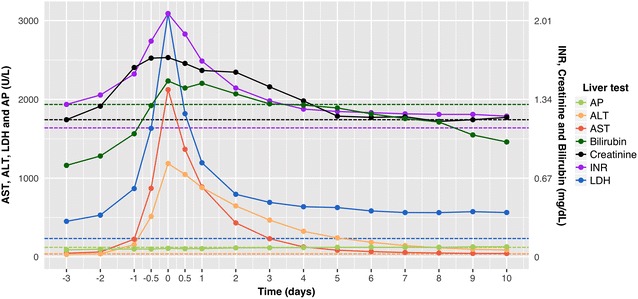
Time trend of liver tests in hypoxic hepatitis of the ASTmax subgroup 5-10x ULN. Time trend of the median AP, ALT, AST, bilirubin, creatinine, INR and LDH levels in hypoxic hepatitis. T-ASTmax is designated as time 0. AST, ALT, LDH and AP levels are measured in U/L and are plotted using the left y-axis. Bilirubin and creatinine levels are measured in mg/dL and plotted using the right y-axis. INR has no unit, but is also plotted using the right y-axis. AP alkaline phosphatase, ALT alanine aminotransferase, AST aspartate aminotransferase, INR international normalized ratio, LDH lactate dehydrogenase

**Fig. 6:**
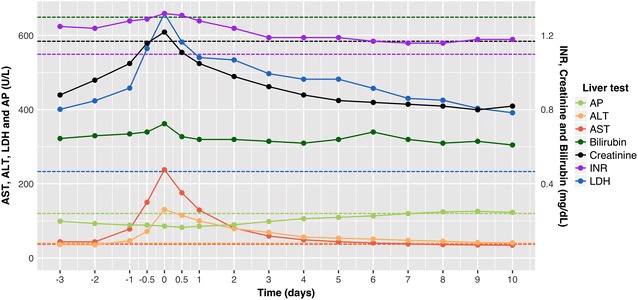
Time trend of liver tests in hypoxic hepatitis of the ASTmax subgroup 10-20x ULN. Time trend of the median AP, ALT, AST, bilirubin, creatinine, INR and LDH levels in hypoxic hepatitis. T-ASTmax is designated as time 0. AST, ALT, LDH and AP levels are measured in U/L and are plotted using the left y-axis. Bilirubin and creatinine levels are measured in mg/dL and plotted using the right y-axis. INR has no unit, but is also plotted using the right y-axis. AP alkaline phosphatase, ALT alanine aminotransferase, AST aspartate aminotransferase, INR international normalized ratio, LDH lactate dehydrogenase

**Fig. 7:**
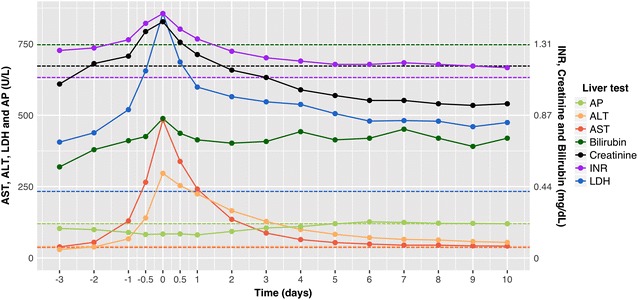
Time trend of liver tests in hypoxic hepatitis of the ASTmax subgroup >20x ULN. Time trend of the median AP, ALT, AST, bilirubin, creatinine, INR and LDH levels in hypoxic hepatitis. T-ASTmax is designated as time 0. AST, ALT, LDH and AP levels are measured in U/L and are plotted using the left y-axis. Bilirubin and creatinine levels are measured in mg/dL and plotted using the right y-axis. INR has no unit, but is also plotted using the right y-axis. AP alkaline phosphatase, ALT alanine aminotransferase, AST aspartate aminotransferase, INR international normalized ratio, LDH lactate dehydrogenase

## Abbreviations

HH: hypoxic hepatitis
AST: aspartate aminotransferase
ICU: intensive care unit
ALT: alanine aminotransferase
LDH: lactate dehydrogenase
ULN: upper limit of normal
CK: creatine kinase
AP: alkaline phosphatase
INR: international normalized ratio
BMI: body mass index
APS-II: acute physiology II
SAPS-II: simplified acute physiology II
T-ASTmax: time point of maximum AST value
ASTmax: maximum AST value
Q1: first quartile
Q3: third quartile
MIMIC-III: Medical Information Mart for Intensive Care III
